# Ilizarov ring fixator in the management of infected non-unions of tibia

**DOI:** 10.1051/sicotj/2015022

**Published:** 2015-07-29

**Authors:** Naveed Bashir Wani, Basit Syed

**Affiliations:** 1 MS (Orthopaedics), Registrar department of orthopaedics, Government Medical College Srinagar J&K India; 2 PG scholar department of orthopaedics, Government Medical College Srinagar J&K India

**Keywords:** Ilizarov, Infected, Non-unions, Tibia

## Abstract

*Purpose*: The purpose of the study was to evaluate the effectiveness of debridement and application of Ilizarov ring fixator (IRF) in the management of infected tibial non-unions.

*Patients and methods*: Twenty six patients with infected non-unions of tibia were managed by debridement and resection of infected portion ± partial fibulectomy and stabilization by Ilizarov ring fixator. Bone segment transport was done in 18 patients who had greater than 2.5 cm bone defect after debridement. Bone grafting was required in three patients to augment union.

*Results*: All fractures united and infection eradicated completely. There were 13 excellent, nine good, and four fair results. Functional results were excellent in nine, good in 11, fair in five and poor in one. Pin site inflammation was the most common problem and occurred in 23 (88%) patients. There were no major complications or neurovascular complications.

*Conclusion*: We conclude that debridement combined with Ilizarov ring fixator with or without partial fibulectomy is a reliable method of treatment of infected non-unions of tibia.

## Introduction

Non-union, particularly infected non-union, is one of the most challenging problems faced by an orthopaedic surgeon. Failure of union may be due to an inappropriate mechanical environment or due to infection and in some cases there is no apparent reason [1]. The prevalence of non-union in closed tibial fractures is 2.5% and it increases five to seven fold for open fractures with gross contamination and extensive soft-tissue damage [2]. Associated fibular fractures usually heal quickly and prevent compression at the fracture site of the tibia thus adversely affecting its healing [3–5]. When lengthening or compression at the non-union site is planned, fibulectomy is a must otherwise an intact fibula contributes to the stability of the non-union [6]. Various options for dealing with infected non-unions are; extensive debridement and local soft tissue rotational flaps, packing the defect with antibiotic impregnated beads, papineau-type open cancellous bone grafting, tibio-fibular synostosis, cancellous allograft in fibrin sealant mixed with antibiotics, and/or free microvascular soft tissue and bone transplants [7]. When non-union, infection, shortening, deformity and osteoporosis occur simultaneously, none of the previously mentioned techniques addresses the above-mentioned problems while allowing weight bearing during the course of treatment. The Ilizarov technique relies on distraction osteogenesis and is used not only for segmental defects, but also to correct complex malalignments with minimal surgery and to overcome shortening and joint contractures by gradual stretching of soft tissues. It can also stimulate bone repair in the most quiescent non-unions, often by distraction alone [8–11]. We have been using Ilizarov technique at our institute for deformity correction, lengthening [12], compound fractures [13] and non-unions for more than a decade. The purpose of this study was to evaluate the efficiency, complications, merits and demerits if any of Ilizarov technique in dealing with infected non-unions of the tibia.

## Materials and methods

This study was carried out on 26 patients with infected tibial non-union at our hospital, between June 2010 and June 2012, after due permission from the Ethics Committee. Patients with clinical and radiological evidence of non-union along with a draining sinus were included. Though six months is the minimum time duration from injury after which a fracture can be considered as non-union, one patient with a grossly infected wound and a frankly mobile fracture site of only five months duration was also included. Patients with associated neuro-vascular injuries or any other condition which would interfere with post-operative rehabilitation were excluded from the study. There were 22 (85%) males and four (15%) females. The average age of the patients was 39 years (20–65). Twelve (46%) non-unions involved the middle third of tibia while eight (31%) involved the lower third. The upper third was involved in six (23%) cases. Eighteen cases were caused by road traffic accidents, four by firearm injuries, one by a fall from height and three followed surgical interventions for a closed fracture. Initially 23 were open (Gustilo Anderson type II 5, III A 6 and III B 12) and three were closed. Pre-operative cultures showed *Staphylococcus Aureus* in 15, pseudomonas in seven, Klebsiella in three and *E. Coli* in one. Mixed flora was seen in eight cases. An average of 2.5 surgical interventions (range 1–5) were carried out in all cases prior to applying an Ilizarov Ring Fixator (IRF) (Table 1).

**Table 1.** Different surgeries done prior to applying IRF.

Surgical procedure	No. of patients
External fixator	24
Debridement and I/ds	20
Bone grafting	5
Skin grafting	5
Plating	6
IMN	4

The IRF was applied after an average of 35.5 weeks (20–120 weeks). Monofocal treatment was used in eight (31%) while bifocal was used in 18 (69%) cases. Average bone segment transported was 5.1 cm in bifocal cases (3–8). Average time in frame in monofocal cases was 23.5 weeks while in bifocal cases it was 78 weeks.

**Figure 1.**
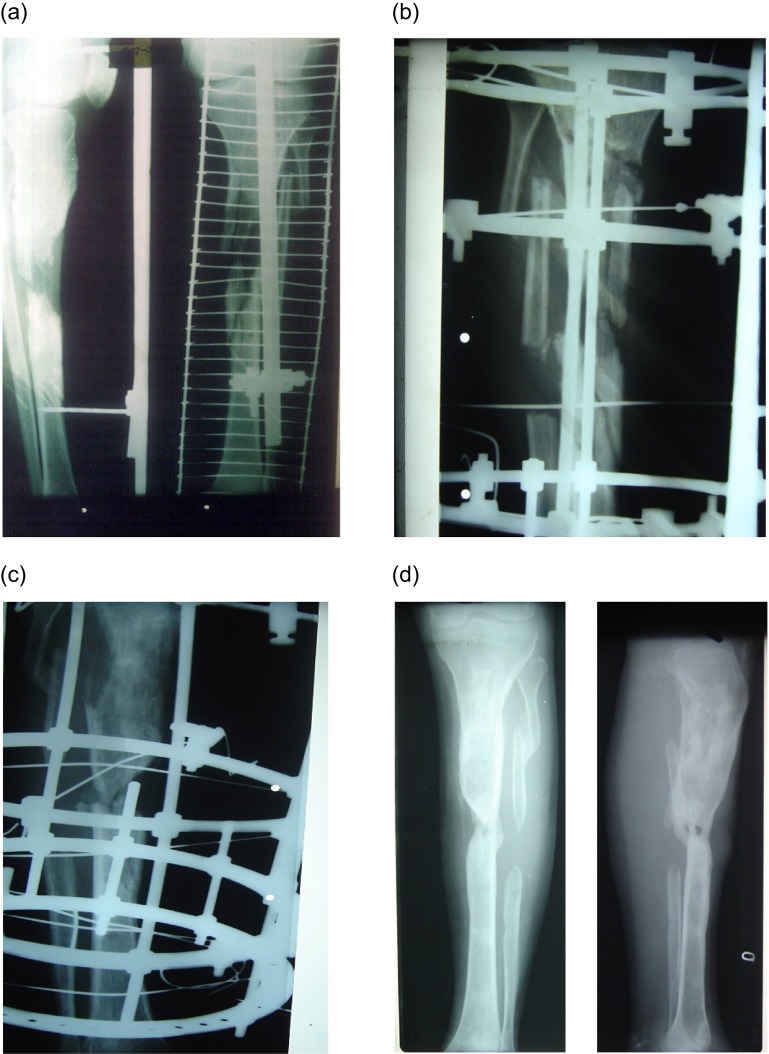
(a) AP and lateral pre-op X-rays showing Frank non-union with bone loss, (b) After applying IRF, corticotomy done at proximal tibia, (c) while undergoing distraction, (d) A/P and lateral views after union.

All patients had their operation under spinal/regional anaesthesia. Pre-operative radiological and clinical findings guided our frame construct (Figure 1a). The non-union site was debrided and freshened and any sequestrate bone or hardware removed. In cases with intramedullary nails, the canal was reamed and thoroughly lavaged after removing the nail. The defect created after freshening and debridement was assessed. If the defect was <2.5 cm (including prior bone loss) monofocal treatment was used, otherwise bifocal treatment was used (Figure 1b). We usually used a 160 mm, 4/5 ring construct but modified it according to need. In lower third fractures of the tibia foot plates were used to prevent equinus deformity. 1.8 mm Ilizarov wires were used. Only pure frames were used. Fibulectomy was done at the junction of middle 1/3rd and lower 1/3rd, but in cases where non-union was at the same site, fibulectomy was done at a higher site. In cases where fibula was fractured in such a way that it would not interfere with tibial union, it was left alone. In the immediate post-operative period, the operated limb was elevated and the distal neuro-vascular status (DNVS) checked. Antibiotics were used according to culture sensitivity report. We did not use pre-operative antibiotics except for the morning dose on the day of surgery. Usually I/V antibiotics were used for three to five days postoperatively with few exceptions (range 3–40 days). Cefazolin and amikacin were the most commonly used antibiotics. On the first post-operative day, frame stability, DNVS and pin sites were checked. Frame stability was checked manually by ensuring that all nut bolts were tightly fitted and wires were properly tensioned. The routine of checking pin sites and frame stability on first post-operative day conveyed to patients/attendants its importance. Gentle range of motion exercises of adjacent joints was started. Patients were encouraged to bear weight on the second post-operative day. Distraction of 1 mm per day was started usually on the 7th post-operative day (range 7–9 day). Patients were discharged on the fifth to tenth post-operative day after teaching them distraction and open pin site care. Patients were encouraged to have a bath and clean the pin sites with soap and water daily.

At follow-ups, frame stability, pin site condition and range of motion of adjacent joints were checked and any complication noted and treated. Radiographs were taken at appropriate times to access union and/or quality of regenerate (Figure 1c). Three patients who did not show any signs of union at the docking site in serial X-rays were bone grafted to augment union at the docking site. We used the posterior-lateral approach for bone grafting. This was harvested from the iliac crests. Temporary frame dismounting was required in two of these cases.

Pin tract inflammation was the most common complication and was graded according to Dahl’s [14] grading and dealt with accordingly:

Grade I normal pin site.
Grade II inflamed.
Grade III inflamed with serous discharge.
Grade IV inflamed with purulent discharge.
Grade V inflamed with osteolysis.
Grade VI inflamed with ring sequestrum.

For grades II and III local care was sufficient and for grade IV systemic antibiotics were given. For grade V we changed the wire. We did not encounter grade VI inflammation. Fracture union and quality of regenerate were assessed by taking X-rays at appropriate times. The quality of regenerate on X-rays was assessed on the basis of Fernandez Esteve [15] grading:

Grade I empty space between two fragments without radiopacity.
Grade II presence of cloud of bony callus.
Grade III presence of periosteal bridge in at least one diaphyseal wall in every X-ray projection.
Grade IV presence of periosteal bridge in both diaphyseal walls in every X-ray projection.
Grade V structural callus is seen.

At fracture site or docking site, the condition of callus and disappearance of fracture lines were looked for. The frame was removed when grade V regenerate was formed and the fracture site and docking site showed signs of union.

## Results

Evaluation of clinical follow-up results was done according to the protocol of Association for the Study and Application of the Method of Ilizarov (ASAMI) [16].

An excellent result was defined as union, no infection, deformity less than 7°, and leg-length inequality of less than 2.5 cm; a good result, as union plus any two of the other three criteria; a fair result as union plus any one of the other criteria; and a poor result as union but none of the other three criteria, or non-union or re-fracture. The functional results were based on five criteria: a noteworthy limp, stiffness of either the knee or the ankle (a loss of more than 15° of full extension of the knee or of 15° of extension (dorsiflexion) of the ankle in comparison with normal contralateral ankle, soft-tissue sympathetic dystrophy, pain that reduced activity or disturbed sleep, inactivity or inability to return to daily activities due to injury.

All 26 patients in this study achieved union of the tibia and any infection eradicated with no sign of recurrence at the last follow-up, which was at least one year after union. We had seven excellent, seven good and four fair results in the bifocal group. In the monofocal group, we had six excellent, one good and one fair result. The functional results in the bifocal group were four excellent, eight good, five fair and one poor. In the monofocal group, there were five excellent and three good results. The patients who needed grafting cannot be considered as excellent as per the ASAMI protocol. A clear difference between results in monofocal and bifocal groups is expected considering the patients treated. Healing index, which is the time duration in frame per centimetre of bone transported, was 1.3–2 months/cm (average 1.6) (Table 2).

**Table 2.** Results.

	Results	
	Bifocal group	Monofocal group
Bone results		
Excellent	7 (39%)	6 (75%)
Good	7 (39%)	1 (12.5%)
Fair	4 (22%)	1 (12.5%)
Functional results		
Excellent	4 (22%)	5 (62%)
Good	8 (44%)	3 (28%)
Fair	5 (28%)	
Poor	1 (6%)	

## Complications

**Figure 2.**
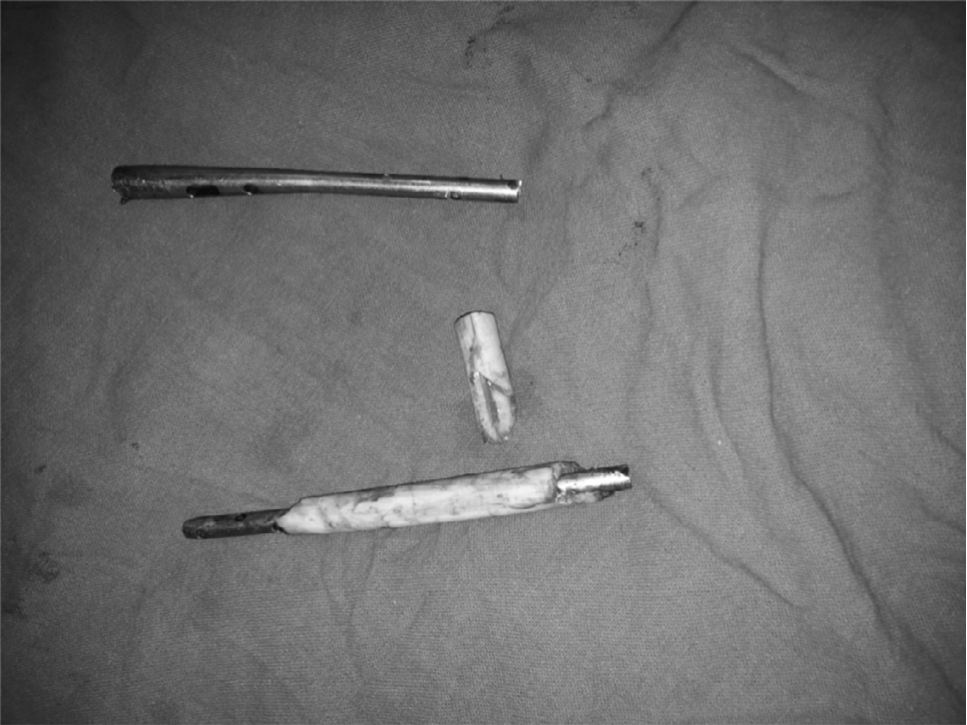
Sequestrum over a nail.

Pin site inflammation was the most common complication that we faced. It occurred in 23 (88%) patients at some stage of the treatment. Two had a local abscess and one had a loose wire secondary to infection. Three patients had delayed consolidation at the docking site. Two patients who had a prolonged time in the frame suffered from depression and were managed with antidepressants. They improved after removal of the frame. Though most of the patients had some degree of joint stiffness at the time of removal of the frame, most improved with physiotherapy. Eight patients had joint stiffness (knee/ankle), four shortening >2.5 cm and nine varus/valgus angulations >7° at the end of treatment. We faced an unusual difficulty in removing a broken intramedullary nail due to a sequestrum covering the nail circumferentially (Figure 2; Table 3).

**Table 3.** Complications.

Complication	Number of patients
Pin tract infection	23 (88%)
Grade 2	15
Grade 3	5
Grade 4	2
Grade 5	1
Restricted ROM*	8 (30%)
Ankle	3
Angulations*	9 (34%)
Varus 3	
Valgus	6
Shortening*	4 (15%)
Depression	2 (7.5%)
Delayed consolidation at docking site	3 (11.5%)
Difficulty in removing hardware	1

ROM: range of motion.

* As per ASAMI criteria.

## Discussion

Infected non-union of long bones presents a challenge to the treating doctor. Most fractures fail to achieve union as a result of damage from initial injury and mechanical instability, which are further compounded by osteomyelitis, bone loss, multiple surgical procedures, disuse osteoporosis, soft-tissue atrophy, decreased arterial blood flow, and impaired venous and lymphatic drainage [17]. Management of these difficult non-unions can be described as limb salvage. The basis of managing an infected non-union is debridement of infected tissues and filling the gap thus created. Bone defect created after debridement of infected necrotic bone can be either filled by vascularised bone graft/simple bone grafting or by osteosynthesis. Vascularised bone graft from fibula or iliac crest can be used but only 40% patients united by such technique in the presence of infection [18]. Autogenous bone graft is limited by availability and cancellous bone takes years to fully corticalise in the presence of infection [19]. Ilizarov distraction osteosynthesis allows resection of the infected bone area, repair of the bone defect, stabilisation of the bone helping it to consolidate while maintaining or restoring the length of the limb as desired. Joint function in the involved extremity is encouraged during the period the apparatus is worn and functional loading can be initiated within the first days after application of frame.

We agree with Schwartzman et al. [6] that fibular osteotomy is always needed when lengthening of the limb is planned or compression of the non-union is to be achieved. Conversely, an intact fibula definitely contributes to the stability of the non-union. Bone grafting is another modality that we can use to stimulate osteogenesis. In cases of recalcitrant non-unions it can be used at the docking site as an adjunct to the Ilizarov method to achieve union or even to shorten the time of treatment.

PTI was the most common complication that we faced as it is with most of the other studies [20]. We used open pin site care in our patients. The reason for advising open pin site care was that it is easier to follow than occlusive one, particularly in our setting where proper nursing and material may not be available to everyone. The higher incidence of PTI in our study may be either due to observational bias as it is difficult to distinguish between grade II and grade III PTI, or due to lower socio-economic and educational level of our patients and as such insufficient pin site care. It leads to loosening of wires and instability of the frame. Pain caused by a loose inflamed wire is an important reason for non-weight bearing and the very purpose of ring fixation is compromised. A proper technique of wire insertion and meticulous post-operative pin site care is very important. Two wire sites developed local abscesses and needed incision drainage. One wire, which loosened due to osteolysis, was replaced. We did not encounter any ring sequestrum.

The healing index was 1.3–2 months/cm (average 1.6). It is similar to the author’s observation in compound fractures [13] and lengthening [12] done at our institute.

While judging results in such a study, the severity of the patient condition and the available options have to be kept in mind. We achieved 100% success rate in eradicating the infections in our patients. Union at the fracture site was achieved in all patients, two (11%) bifocal and one (12.5%) of the monofocal cases needed augmentation by bone grafting at the docking site to achieve union. This higher percentage in monofocal group may be due to a lower number of cases in this group. All three were smokers who smoked even during the treatment period. Similar observations have been made by others [21]. Though we believe in Ilizarov’s assertion that distraction alone is a potent stimulus for union [11], we also believe that bone grafting, particularly in atrophic non-unions, is a viable option for reducing the duration in frame. Green grafted the docking site while Ilizarov freshened it with curette and osteotome. Our overall bone results were excellent in 13 (50%), good in eight (31%) and fair in five (19%). Functional results were excellent in nine (35%), good in 11 (42%), fair in five (19%) and poor in one (4%). When we compare our results with other studies, Dendrinos et al. [22] had in a study of 28 infected tibial non-unions 14 (50%) excellent. Eight (28.5%) good, one (3.5%) fair and five (18%) poor results. Mugadum et al. [23] in a study of 25 infected tibial non-unions had 19 (76%) excellent, five (20%) good and one (4%) poor while functional results excellent in 15 (60%), good in eight (32%), one fair (4%) and one poor (4%). These results were comparable to our results. We had excellent or good results in all eight patients in the monofocal group while in the bifocal group 12 (66%) had excellent or good results. The obvious difference between monofocal and bifocal group can be explained on the basis of severity of the case and complications associated with bone transport. All these patients were satisfied with the treatment. Five patients had fair and one had a poor functional result. We had no nerve or vessel injuries. Keeping in view the complexity of the problems we dealt with, we feel satisfied with the treatment option and recommend its use in such cases with the caution that it requires patience on the part of both patients and treating doctors.

## Conflict of interest

No funding either directly or indirectly has been received by any of the authors for this study.
